# An atypical case of choroidal neovascularization associated with pseudoxanthoma elasticum treated with intravitreal bevacizumab: a case report

**DOI:** 10.1186/1756-0500-6-530

**Published:** 2013-12-11

**Authors:** Michael Karampelas, Vasileios Soumplis, Dimitrios Karagiannis, Efstratios Parikakis, Andrew R Webster

**Affiliations:** 1 Moorfields Eye Hospital, 162 City Road, London EC1V 2PD, UK; 2 Ophthalmiatrion Eye Hospital, NHS, Athens, Greece; 3 Institute of Ophthalmology, University College London, London, UK

**Keywords:** Bevacizumab, Choroidal neovascularization, Pseudoxanthoma elasticum

## Abstract

**Background:**

Pseudoxanthoma elasticum is an inherited disorder that is associated with accumulation of pathologic elastic fibers in the skin, vascular walls and Bruch’s membrane in the eye. Choroidal neovascularization is one of the most common causes of acute vision loss in these patients. We report an atypical case of suspected choroidal neovascularization associated with pseudoxanthoma elasticum.

**Case presentation:**

A 47-year-old Caucasian woman with pseudoxanthoma elasticum and angioid streaks was referred because of decreased and distorted vision in her right eye of one week’s duration. Visual acuity was 6/12 in the right eye and 6/6 in the left eye. Fundus examination revealed angioid streaks and white intraretinal macular deposits bilaterally. Fluorescein angiography did not reveal any obvious leakage in the right eye while optical coherence tomography revealed subretinal fluid associated with an adjacent intraretinal hyperreflective structure. Autofluoresence imaging showed focal areas of increased autofluorescence corresponding to the deposits in both eyes. Over the following year the patient underwent five intravitreal injections of bevacizumab (Genentech/Roche,US) in the right eye, which resulted in visual acuity improving to 6/9 with regression of the hyperreflective structrure and complete resolution of subretinal fluid.

**Conclusions:**

Traditionally, fluorescein angiography is effective in the detection of choroidal neovascularization in patients with pseudoxanthoma elasticum. In our case, optical coherence tomography revealed subretinal fluid and an adjacent hyperreflective structure while fluorescein angiography did not reveal any obvious leakage. The sole presence of subretinal fluid does not necessarily imply the presence of choroidal neovascularization and certainly retinal pigment epithelium dysfunction could also explain subretinal fluid in these patients. However, the complete absorption of the fluid and the disappearance of the previously evident hyperreflective structure following treatment, led us to suspect choroidal neovascularization as the primary cause of the above findings. The poor natural course of choroidal neovascularization in these patients increases the importance of early detection and should result in the adaptation of a low-threshold strategy concerning the initiation of treatment.

## Background

Pseudoxanthoma elasticum (PXE) is an inherited disorder that is associated with accumulation of mineralized and fragmented elastic fibers in the skin, vascular walls and Bruch’s membrane in the eye [1]. *ABCC6*, an ATPbinding cassette transporter gene encoding a multidrug resistance protein (*MRP6*), had been identified as the defective gene [2]. Ocular manifestations include angioid streaks, peau de orange, optic disc drusen and comet lesions [3]. Choroidal neovascularization (CNV) is one of the most common causes of acute vision loss in PXE patients [4]. We report the presentation and management of a case of CNV in a patient with PXE in whom there was absence of obvious leakage during fluorescein angiography (FA) and the presence of subretinal fluid (SRF) as well as an intraretinal hyper-reflective structure in spectral-domain optical coherence tomography (SD-OCT).

## Case presentation

**Figure 1:**
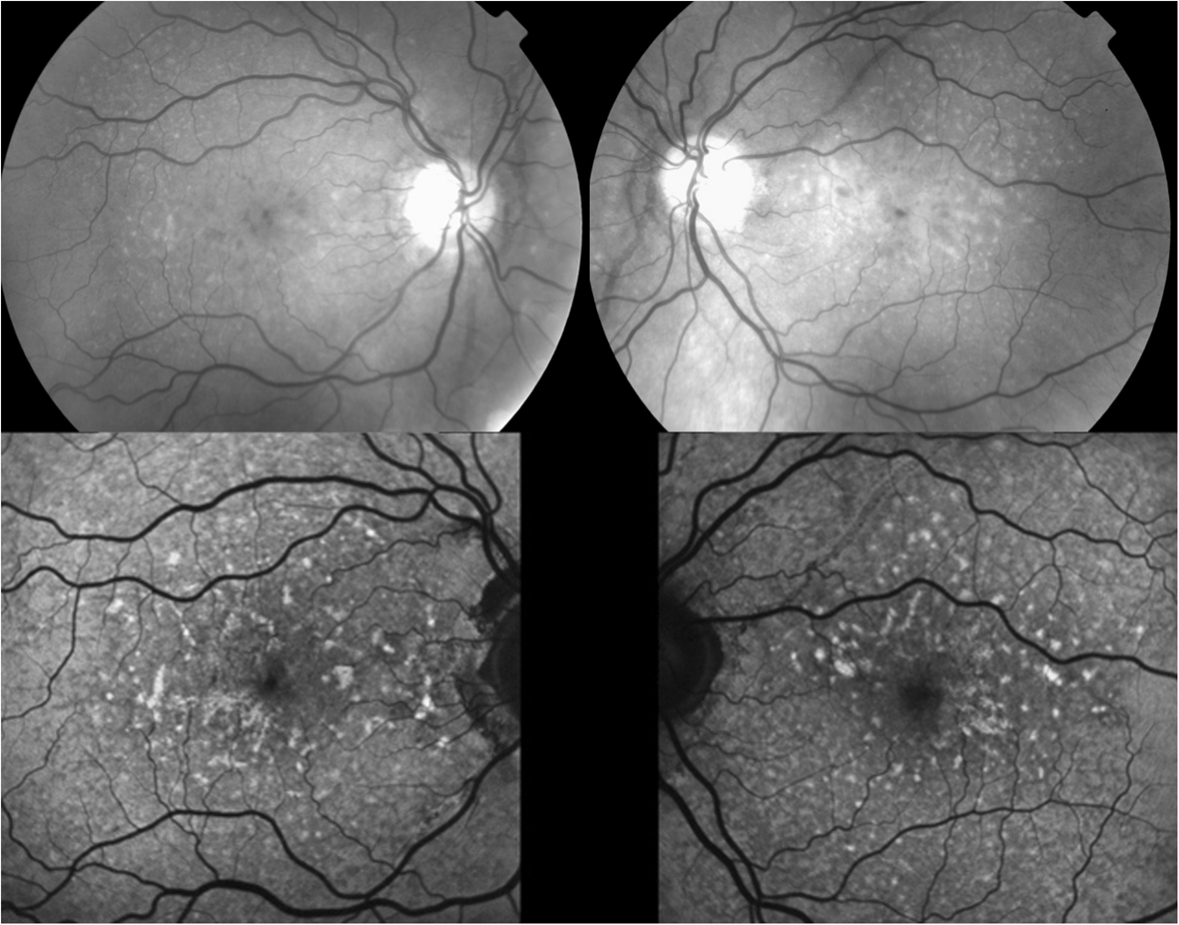
**Red-free and autofluoresence images.** Red-free photographs showing angioid streaks and intraretinal macular deposits bilaterally and fundus autofluoresence images showing focal areas of increased autofluorescence.

**Figure 2:**
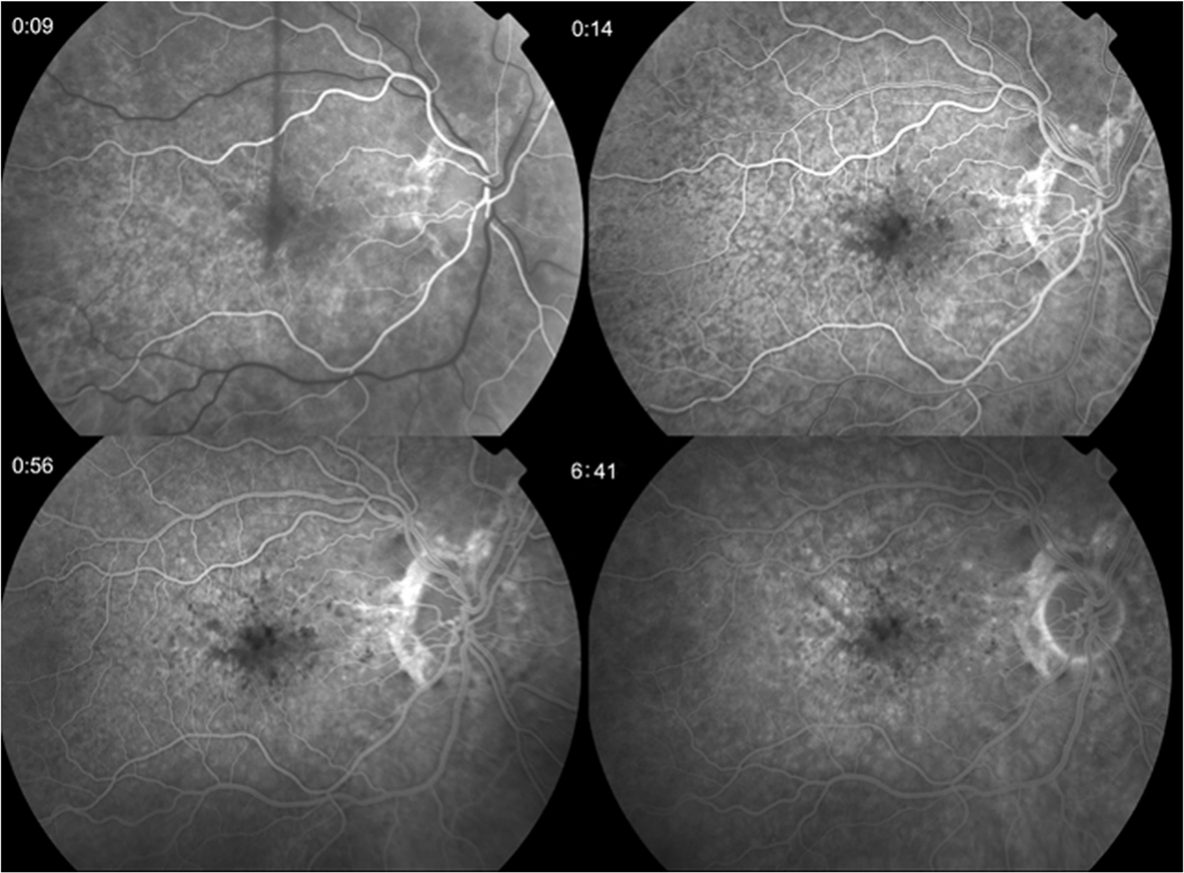
Fluorescein angiography of the right eye at presentation showing no obvious leakage.

**Figure 3:**
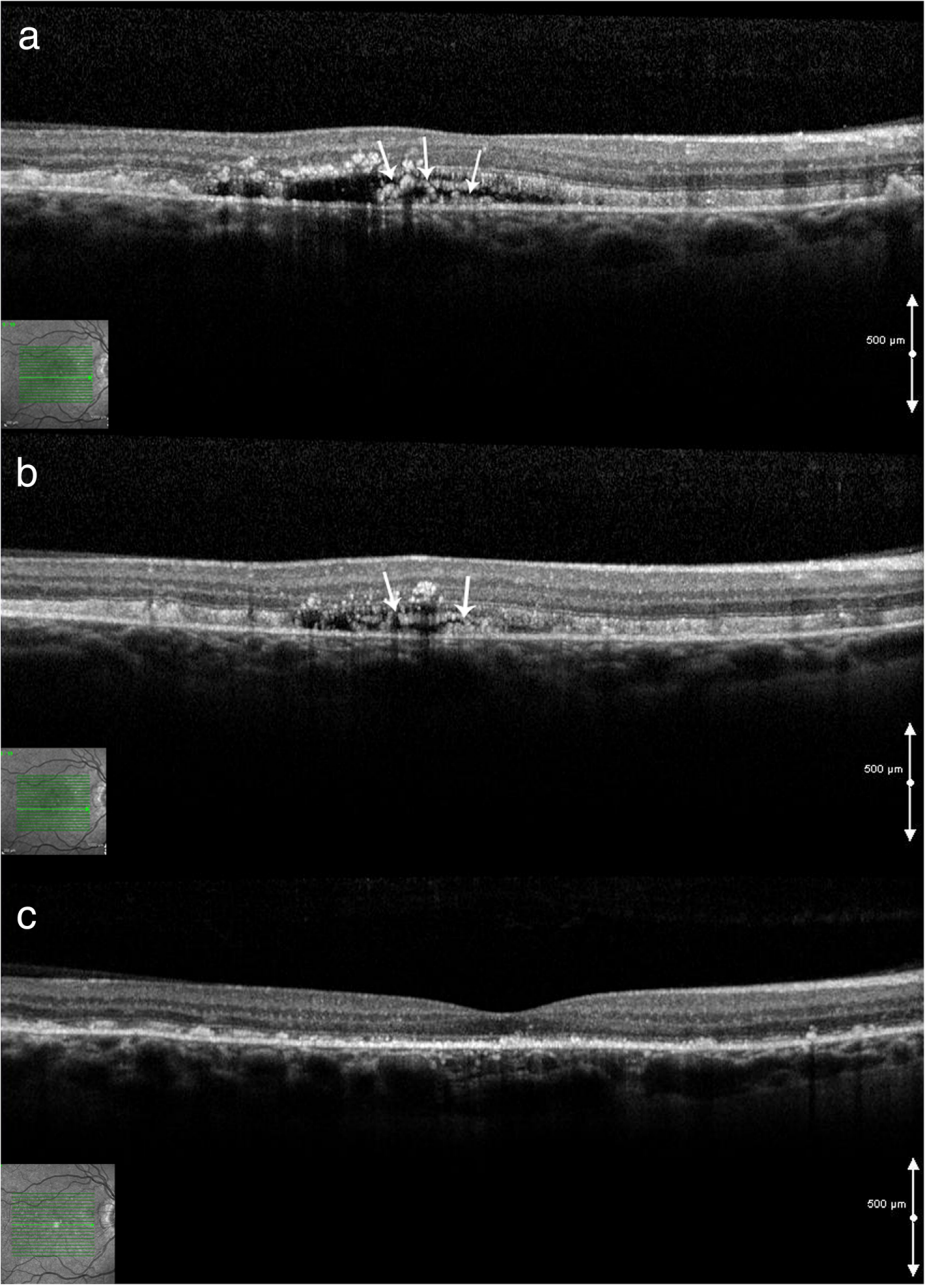
**Spectral-domain optical coherence tomography at presentation and post treatment. a,b)** Spectral-domain optical coherence tomography of the right eye at *presentation showing* subretinal fluid and contiguous intraretinal hyperreflective material (white arrows) (two different horizontal scans are shown) **c)** Spectral-domain optical coherence tomography of the *right eye* after 5 intravitreal injections of bevacizumab showing resolution of subretinal fluid and absence of the hyperreflective structure.

**Figure 4:**
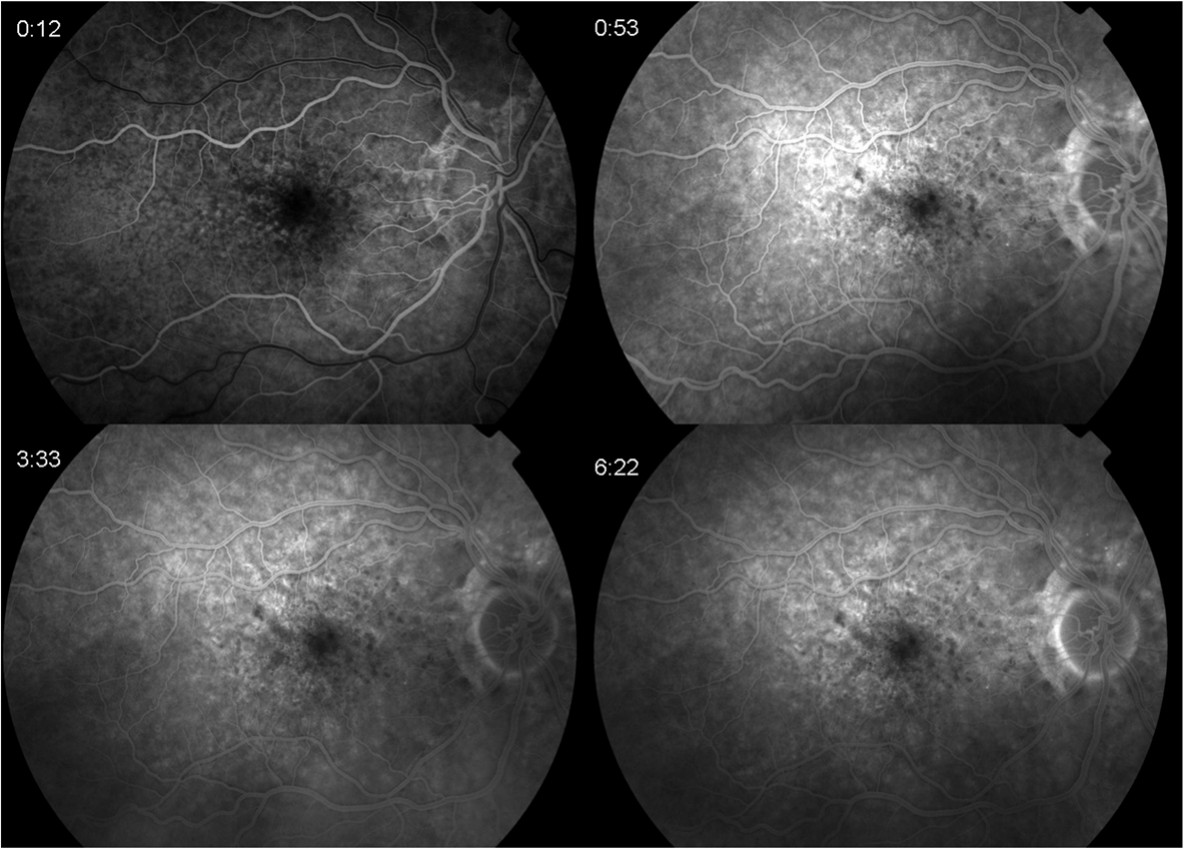
Fluorescein angiography of the right eye one year after presentation with similar appearance as presentation.

A 47 year old Caucasian woman with PXE and angioid streaks was referred because of decreased and distorted vision in her right eye (RE) of one week’s duration. At presentation visual acuity (VA) was 6/12 in the RE and 6/6 in the LE. Fundus examination revealed angioid streaks and white intraretinal macular deposits bilaterally (Figure 1). FA did not reveal any obvious leakage (Figure 2). Contemporaneous SD-OCT revealed SRF associated with a contiguous hyperreflective structure suggestive of a Type II CNV, as well as generalized thickening and hyperreflectivity of the outer retina between the photoreceptor inner/outer segments and retinal pigment epithelium (RPE) bands (Figure 3a,b). Indocyanine angiography was not performed since the pathology appeared to be intraretinal and there was no obvious masking of the choroidal circulation by blood or pigment. Fundus autofluoresence showed focal areas of increased autofluorescence in both eyes (Figure 1). We obtained informed consent and the patient underwent five monthly intravitreal injections of bevacizumab (Genentech/Roche,US) in the RE. After one year of follow up the fluid did not reappear, and VA improved to 6/9 with regression of the membrane and resolution of SRF (Figure 3c). FA at that stage did not reveal any significant change compared to baseline (Figure 4).

## Conclusions

CNV occurs in 72% to 86% of eyes with angioid streaks and can lead to dramatic visual impairment [4]. Traditionally, FA is effective in the detection of CNV in patients with PXE, but in some cases prompt diagnosis could be challenging due to the subtlety or absence of related signs. In the case presented herein, SD-OCT revealed SRF and a hyperreflective structure that led to the suspicion of a Type II CNV. The classic angiographic appearance of Type II CNV is that of early hyperfluorescence [5], and therefore the absence of any obvious leakage in the FA was an unexpected finding.

Zweifel et al. recently reported the presence of SRF not associated with PXE [6]. The authors concluded that the observed SRF was not associated with CNV, due to the complete lack of response to intravitreal anti-vascular endothelial growth factor (VEGF). In contrast to their findings, in our case SRF responded to anti-VEGF treatment. Certainly, another pathogenetic mechanism for the presence of SRF in these patients could be retinal pigment epithelium dysfunction, similarly to chronic serous chorioretinopathy (CSR). However, anti-VEGF agents have not been proven to be efficacious in CSR [7,8] and therefore the complete absorption of the SRF and the disappearance of the previously evident hyperreflective structure following anti-VEGF treatment in our case, led us to suspect CNV as the primary cause of the above findings.

The effectiveness of anti-VEGF treatment for CNV secondary to PXE has been reported in numerous studies [4,9-14]. Data from these studies indicate that CNV in these patients is associated with multiple recurrences and that early treatment seems to be of great importance. Regular monitoring for long periods is recommended as well as frequent reinjections at the slightest signs of CNV activity [11]. Given the subtly of signs in our case and the heightened sensitivity of SD-OCT, we would recommend the careful investigation with SD-OCT in the management of these cases. The poor natural course of CNV in these patients [15] increases the importance of early detection and should result in the adaptation of a low-threshold strategy concerning the initiation of treatment. Our case demonstrates the importance of SD-OCT in patients with clinical signs suggesting CNV without corresponding FA findings.

## Consent

Written informed consent was obtained from the patient for publication of this Case Report and any accompanying images. A copy of the written consent is available for review by the Editor-in-Chief of this journal. This study has been reviewed by our institute ethics committee and therefore was performed in accordance with the ethical standards laid down in the 1964 Declaration of Helsinki.

## Abbreviations

PXE: Pseudoxanthoma elasticum; CNV: Choroidal neovascularization; FA: Fluorescein angiography; SRF: Subretinal fluid; SD-OCT: Spectral-domain optical coherence tomography; RE: Right eye; VA: Visual acuity; RPE: Retinal pigment epithelium; VEGF: Vascular endothelial growth factor.

## Competing interests

The authors declare that they have no competing interests.

## Authors’ contributions

MK and VS examined and treated the patient and in doing so acquired the case data; they were also involved with drafting of the manuscript. DK, EP and AW participated in its design and coordination and helped to draft and revise the manuscript. All authors read and approved the final manuscript.
